# Analysis of urine Raman spectra differences from patients with diabetes mellitus and renal pathologies

**DOI:** 10.7717/peerj.14879

**Published:** 2023-02-27

**Authors:** Varun Kavuru, Ryan S. Senger, John L. Robertson, Devasmita Choudhury

**Affiliations:** 1 Virginia Tech Carilion School of Medicine, Roanoke, VA, United States; 2 University Hospital at University of Virginia Medical Center, Charlottesville, VA, United States; 3 Department of Biological Systems Engineering, Virginia Polytechnic Institute and State University (Virginia Tech), Blacksburg, VA, United States; 4 DialySensors, Inc., Blacksburg, VA, United States; 5 Department of Biomedical Engineering and Mechanics, Virginia Polytechnic Institute and State University (Virginia Tech), Blacksburg, VA, United States; 6 Salem Veteran Affairs Health Care System, Salem, VA, United States

**Keywords:** Raman spectroscopy, Chronic kidney disease, Diabetes mellitus, Diabetic kidney disease, Chemometric analysis, Immune mediated kidney disease, Discriminant analysis, Urine

## Abstract

**Background:**

Chronic kidney disease (CKD) poses a major public health burden. Diabetes mellitus (DM) is one of the major causes of CKD. In patients with DM, it can be difficult to differentiate diabetic kidney disease (DKD) from other causes of glomerular damage; it should not be assumed that all DM patients with decreased eGFR and/or proteinuria have DKD. Renal biopsy is the standard for definitive diagnosis, but other less invasive methods may provide clinical benefit. As previously reported, Raman spectroscopy of CKD patient urine with statistical and chemometric modeling may provide a novel, non-invasive methodology for discriminating between renal pathologies.

**Methods:**

Urine samples were collected from renal biopsied and non-biopsied patients presenting with CKD secondary to DM and non-diabetic kidney disease. Samples were analyzed by Raman spectroscopy, baselined with the ISREA algorithm, and subjected to chemometric modeling. Leave-one-out cross-validation was used to assess the predictive capabilities of the model.

**Results:**

This proof-of-concept study consisted of 263 samples, including renal biopsied, non-biopsied diabetic and non-diabetic CKD patients, healthy volunteers, and the Surine™ urinalysis control. Urine samples of DKD patients and those with immune-mediated nephropathy (IMN) were distinguished from one another with 82% sensitivity, specificity, positive-predictive value (PPV), and negative-predictive value (NPV). Among urine samples from all biopsied CKD patients, renal neoplasia was identified in urine with 100% sensitivity, specificity, PPV, and NPV, and membranous nephropathy was identified with 66.7% sensitivity, 96.4% specificity, 80.0% PPV, and 93.1% NPV. Finally, DKD was identified among a population of 150 patient urine samples containing biopsy-confirmed DKD, other biopsy-confirmed glomerular pathologies, un-biopsied non-diabetic CKD patients (no DKD), healthy volunteers, and Surine™ with 36.4% sensitivity, 97.8% specificity, 57.1% PPV, and 95.1% NPV. The model was used to screen un-biopsied diabetic CKD patients and identified DKD in more than 8% of this population. IMN in diabetic patients was identified among a similarly sized and diverse population with 83.3% sensitivity, 97.7% specificity, 62.5% PPV, and 99.2% NPV. Finally, IMN in non-diabetic patients was identified with 50.0% sensitivity, 99.4% specificity, 75.0% PPV, and 98.3% NPV.

**Conclusions:**

Raman spectroscopy of urine with chemometric analysis may be able to differentiate between DKD, IMN, and other glomerular diseases. Future work will further characterize CKD stages and glomerular pathology, while assessing and controlling for differences in factors such as comorbidities, disease severity, and other lab parameters.

## Introduction

It is estimated that the prevalence of chronic kidney disease (CKD) in the United States (US) is approximately 14.9% and represents a significant financial burden. In 2018, Medicare expenditures for patients with CKD without end stage renal disease (ESRD) exceeded $81 billion and expenditures for patients with ESRD rose above $49 billion (USRDS, 2021). Nearly 40% of diabetic patients ultimately develop renal disease, and diabetic kidney disease (DKD) is the leading cause of ESRD in the US (Piccoli et al., 2015; Thomas et al., 2015; Erdogmus et al., 2017; Krolewski et al., 2017; Centers for Disease Control and Prevention (CDC), 2020).

Renal biopsy-diagnosed DKD and diabetic nephropathy (DN) are terms commonly used to describe dysfunction of the kidneys as a result of the diabetic disease process (Santoro et al., 2021). Evidence of hyperfiltration, proteinuria, and diffuse glomerular disease with glomerular basement thickening, mesangial cell expansion, accumulation of advanced glycosylation end products (AGEs), and Kimmelstiel-Wilson nodular lesions are hallmarks of DKD. A clinical diagnosis of DKD is commonly made based on history, physical examination, and abnormal laboratory findings (*i.e*., decreased effective glomerular filtration rate (eGFR) and proteinuria). Renal biopsy is considered the gold standard for detecting glomerular pathology associated with DKD. However, biopsy may often be deferred in diabetes mellitus (DM) patients with no other clinical findings (*e.g*., hematuria) suggestive of other types of glomerular disease, and with longstanding DM with proteinuria, and the presence of disseminated microvascular disease (*e.g*., diabetic retinopathy). Renal biopsy is commonly deferred due to expense, availability of skilled physicians or technical staff able to perform and interpret renal biopsies, and risks including bleeding, development of arterio-venous fistulae, infection, and very rarely, the need for embolization and/or nephrectomy to control bleeding (Madaio, 1990; Stratta et al., 2007; Kitterer et al., 2015).

The usual treatment for management of DKD includes optimal blood glucose and blood pressure control, as well as blocking of the renin angiotensin system (RAS) with angiotensin converting enzyme inhibitors (ACEI), receptor blockers (ACE-RB), and aldosterone antagonists (Lim, 2014). However, DM patients can also develop or may initially have glomerular diseases other than DKD, including IgA nephropathy, lupus nephritis, minimal change disease, focal sclerosis, and/or membranous nephropathy. These nephropathies may have similar clinical presentations but require different treatment options, including steroids and immunosuppression. Therefore, renal biopsy becomes necessary for specific diagnosis (Zhou et al., 2008; Moura-Neto & Ronco, 2021).

Routine histopathologic evaluation of renal biopsies with differential staining (hematoxyin-eosin, periodic acid-Schiff reagent) is generally sufficient for making a diagnosis of DKD. More recently, other advanced techniques have been employed, including proteomic investigations of renal biopsy tissue, laser capture microdissection followed by liquid chromatography, and mass spectroscopy on formalin-fixed tissue sections (Satoskar et al., 2012; Hobeika et al., 2017). Pathologic glomerular remodeling and dysfunction may allow unique proteins to pass into the urine (Haas, 2018). Results from gel electrophoresis and mass spectroscopy studies have suggested that characterization of the urine proteome and identification of some specific urine biomarkers can be used to determine the cause and severity of glomerular disease (Varghese et al., 2007; Santucci et al., 2013).

Raman spectroscopy and surface enhanced Raman scattering (SERS) have been used in the analysis of biological fluids, including blood (Berger et al., 1999; Enejder et al., 2002), urine (Premasiri, Clarke & Womble, 2001; Moreira et al., 2017), saliva (Virkler & Lednev, 2010; Hardy et al., 2022), and others with clinical applications (Panikar et al., 2021). Recently, Raman spectroscopy was used to analyze the spectral differences of urine composition from healthy subjects and from patients with advanced kidney disease, including CKD 4/5 and ESRD (Senger et al., 2020; Chen et al., 2020b, 2020a; Gulyamov et al., 2021; Kamińska et al., 2022). These studies found that chemometric analysis of urine Raman spectral data detected molecular ‘fingerprints’ associated with advanced kidney disease (Senger et al., 2019, 2020; Chen et al., 2020b).

Raman spectroscopy was developed as a tool used to investigate the interactions of radiation with matter; it detects the inelastic scattering of light (vibrational/deformational energy) by molecular bonds. Raman spectrometers detect this change and visualize it as a peak on a spectrum that is characteristic of each molecule (Ember et al., 2017; Shen, Hu & Min, 2019; Cialla-May, Schmitt & Popp, 2019). Chemometric and multivariate statistical analyses of Raman spectra have been used to characterize cellular phenotypes, identify pathogens, and detect the presence of disease (Lussier et al., 2020; Senger & Scherr, 2020). Rametrix^®^ is a multi-component computational platform that combines several statistical modeling tools to perform chemometric analyses of Raman spectral datasets (Fisher et al., 2018; Senger & Robertson, 2020).

In this study, we investigated the use of Raman spectroscopy and Rametrix^®^ chemometric analyses on urine from patients with DM- and non-DM-associated CKD in order to evaluate the potential use of this methodology as a non-invasive tool for differentiating those with DKD from other glomerular diseases. We hypothesized that analysis of patient urine by Raman spectroscopy and Rametrix^®^ would be able to distinguish DKD from immune-mediate nephropathy (IMN) with a low misclassification rate (<5%) on samples cross-validated through leave-one-out-analysis. We also hypothesized that Rametrix^®^ would differentiate urine of patients with DKD from those with other glomerular diseases, DM, healthy volunteers, and the urinalysis standard Surine™ with sensitivity/specificity that exceeded random chance assignments. We believe that the use of these non-invasive chemometric urinalysis procedures could potentially reduce the need for invasive procedures such as renal biopsy.

## Materials and Methods

### Research design

This study was conducted with the approval of the Salem Veterans Affairs Health Care System (SVAHCS) Institutional Review Board, under IRB protocol DCD0014. All patients provided informed written consent and then voluntarily produced a voided urine sample (minimum of 25 mL) for analysis. All patients were enrolled as subjects in the Program to Improve Chronic Kidney Education and Outcomes (PICKEDOUT) study, a pragmatic trial aimed at studying the effects of renal disease and interventional dietary education on patient outcomes such as eGFR.

### Study population

Inclusion criteria included patients of the PICKEDOUT study who submitted a urine sample for analysis by Raman spectroscopy in the last 5 years (*n* = 263), including those patients who had a renal biopsy at the SVAHCS showing proven kidney disease (*n* = 34). Although part of the PICKEDOUT study, these patients did not undergo any education or diet randomization and provided only a single baseline urine sample. The patient population at SVAHCS is predominantly elderly (dataset mean age = 68; median age = 70) and male (dataset 92.4% male). This dataset included urine spectra from un-biopsied SVAHCS patients in the PICKEDOUT study: 161 patients with DM (classified as “DM+”) and 65 patients without DM (DM−). In addition, a subset of 25 samples from a published dataset containing urine Raman spectra from healthy volunteers (Senger et al., 2019) (median age 21 and 19% male) were used in this study. The subset was chosen because the same spectrometer used to analyze these samples was used with SVAHCS patients in this study. In addition, 22 samples of synthetic urinalysis standard Surine™ were also included in this study. Surine™ samples were scanned at the same time as patient samples throughout the duration of the study.

### Sample preparation and storage

Urine samples were kept frozen in standard collection cups at −70 °C for up to 28 days, until analyzed. For analysis by Raman spectroscopy, urine samples were thawed to room temperature (25 °C) and transferred to 2 mL borosilicate glass vials (0.78 mm glass thickness) (Thermo Fisher Scientific, Waltham, MA, USA). A previous study has shown this procedure preserves Raman spectral integrity (Huttanus et al., 2020).

### Raman spectroscopy

Samples were analyzed in bulk liquid phase using an Agiltron (Woburn, MA, USA) PeakSeeker PRO-785 Raman spectrometer equipped with liquid sample holder. Raman scans were conducted using a 785 nm laser with 30 mW laser power, 8 cm^−1^ spectral resolution, 0.2 mm laser spot size, 200–2,400 cm^−1^ Raman shift range, and 30 s excitation time. Ten (10) replicate scans were obtained per sample. Spectra were collected and processed with RSIQ software (Agiltron, Woburn, MA, USA). A urine analytical standard, Surine™ (DynaTek, Lenexa, KS, USA), was also scanned as a system and computational control. Surine™ is synthetic urine that has been employed as a control in prior Rametrix™ studies (Senger et al., 2019, 2020; Huttanus et al., 2020).

### Chemometric analysis of Raman spectra

Raman spectra were analyzed by chemometric methods involving principal component analysis (PCA) and discriminant analysis of principal components (DAPC) as described previously (Lever, Krzywinski & Altman, 2017; Fisher et al., 2018; Senger & Robertson, 2020; Senger et al., 2020; Lussier et al., 2020; Huttanus et al., 2020; Senger & Scherr, 2020). Calculations were performed in MATLAB R2018a (MathWorks, Natick, MA, USA) with the Statistics and Machine Learning, Bioinformatics, Rametrix^®^ LITE v1.0, and Rametrix^®^ PRO v1.0 Toolboxes. The Rametrix^®^ toolboxes are shared publicly through GitHub (Fisher et al., 2018; Senger & Robertson, 2020). First, raw spectra were processed by averaging scan replicates, truncating to a Raman shift range of 400–1,800 cm^−1^, baselining, and vector normalization. Averaging scan replicates during these pre-processing steps ensured urine samples were only represented once in the datasets used for statistical modeling. Spectral baselining was performed with the ISREA algorithm (Xu et al., 2021). ISREA connects cubic splines at “nodes” (or “knots”) located along spectra to form the spectral baseline. Eight or fewer nodes were used in this study, and their locations were varied based on the analysis being performed. Processed spectra were then subjected to PCA and the top principal components (PCs) representing 99% of the dataset variance were used in multivariate analysis of variance (MANOVA) and DAPC (also referred to as “PCADA” and “PCA-DA” elsewhere) to cluster samples according to a specified factor (*e.g*., disease state). The criterion to use the number of PCs representing 99% of dataset variance was derived from prior studies (Senger & Robertson, 2020; Huttanus et al., 2020). DAPC models were validated by leave-one-out cross-validation where all samples were left-out of one model-build and their classification predicted. Model predictions were then used to calculate prediction metrics of accuracy, sensitivity, positive-predictive value (PPV), and negative-predictive value (NPV) (Trevethan, 2017). Following cluster analysis, PC and DAPC loadings were used to determine which Raman shifts contributed most to cluster separations. These Raman shifts (*i.e*., bands) were evaluated using spectral databases of biological molecules (Movasaghi, Rehman & Rehman, 2007; Talari et al., 2015) to suggest molecular differences between samples.

## Results

### Patients and samples

Information regarding subjects such as age, sex, clinically relevant laboratory information such as effective glomerular filtration rate, proteinuria, and renal biopsy results were obtained through review of electronic medical records by SVAHCS staff. The datasets were compiled as shown in Table 1. Thirty-four (34) patients had biopsy-confirmed renal pathology, of which 12 were identified to have DKD, 12 to have IMN, and one with both DKD and IMN (Table 1). The specific pathologies associated with the 11 IMN samples included: membranous lupus nephritis (two samples), IgA nephropathy with mesangial hypercellularity (one sample) (overlapping with DKD), IgA nephropathy (two samples), crescentic ANCA (antineutrophil cytoplasmic antibody) glomerulonephritis (one sample), focal necrotizing glomerulonephritis (myeloperoxidase (MPO)-ANCA) (two samples), evidence of immune-mediated glomerulonephritis (one sample), proliferative glomerulonephritis (one sample), focal segmental necrotizing proliferative glomerulonephritis (one sample), and immune complex glomerulopathy (one sample). In addition, other biopsied patients were also found to have membranous nephropathy (MN), renal neoplasia (RN), and other pathologies (OT). The similar MN pathologies were described as: membranous nephropathy (two samples), membranous glomerulonephropathy (two samples), and membranous glomerulonephritis (two samples). The RN pathologies included: clear cell renal carcinoma (two samples) (one sample overlapping with DKD) and urothelial carcinoma (one sample). The pathologies classified as “Other (OT)” in Table 1 included: transplant glomerulopathy (one sample) and other non-specific forms of glomerulopathy (three samples).

**Table 1:** Summary of patient and urine specimen classifications used in this study.

Description	Classification	Number of samples	Source of data
Patients with biopsy confirmed diabetic nephropathy (DKD)	DKD	12	This study
Patients with immune-mediated nephropathy (IMN)	IMN	12	This study
Patients with DM and no biopsy	DM+	161	This study
Patients without DM and no biopsy	DM−	65	This study
DKD patients with IMN	DKD/IMN	1	This study
DKD patients with other varying pathologies	DKD/OT	3	This study
DKD patients with no other pathologies	DKD/NO	9	This study
IMN patients that are DM+	IMN/DM+	6	This study
IMN patients that are DM−	IMN/DM−	6	This study
Patients with membranous nephrology (cause unspecified)	MN	6	This study
Renal neoplasia	RN	3	This study
Other varying pathologies	OT	4	This study
Pathologies with no DM	PATH	9	This study
Healthy volunteers	HEALTHY	25	Huttanus et al. (2020)
Surine™ urinalysis control	SURINE	22	This study

### Processed Raman spectra

**Figure 1:**
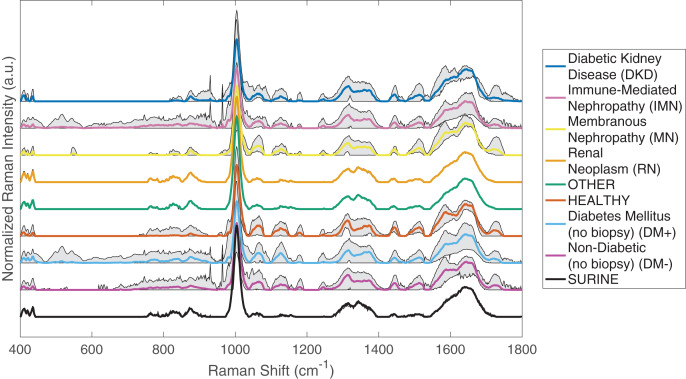
Processed Raman spectra of urine from patients of different disease classes, healthy volunteers, and Surine™. Spectra were averaged, truncated (400–1,800 cm^−1^), and baselined with ISREA. Grey shading represents the range of Raman signal observed at each Raman shift.

Processed urine Raman spectra for the 34 biopsied patients, 161 un-biopsied nephrology patients with diabetes mellitus (DM+), 65 un-biopsied non-diabetic nephrology patients (DM−), 25 healthy volunteers, and 22 spectra of the urinalysis standard Surine™, all obtained at different days/times throughout the study, are shown in Fig. 1. For this analysis, the Raman spectra were baselined with ISREA, using five nodes located at 400, 950, 1,100, 1,475, and 1,800 cm^−1^. Grey shaded regions of Raman spectra plots are indicative of variability observed for the samples analyzed in different Raman shift regions. These are also indicative of urine molecular composition heterogeneity among patients under the given classification (*i.e*., disease).

Surine™ urinalysis analytical control spectra were used to confirm proper calibration and operation of the spectrometer throughout the duration of the study. As shown in Fig. 1, the 22 spectra of Surine™ collected over the 52 weeks of sample collection showed the least amount of variation among all classification groups studied. This also confirms that differences observed between classes and within the same class are due to differences in spectral characteristics and not artifacts of spectrometer operation or measurement. Other classifications showing low intra-class variability were the RN and OT classes. These classes contained three and four samples respectively, and their average spectra resembled one another. Another source of inter- and intra-class variance was observed in the relatively strong band at 1,002 cm^−1^, which is associated with urea in urine studies (Senger et al., 2019, 2020). Visual inspection of the spectra in Fig. 1 identified regions of difference between disease classes, such as between 700–950, 1,000–1,200, 1,250–1,450, 1,500–1,750 cm^−1^ and combinations of these. Of particular interest is the region of 400–800 cm^−1^ for un-biopsied patients with DM+ and those with DKD. Spectral bands in this region appeared to be minimized as DM+ progressed to DKD. This, and other relationships not apparent through visual inspection, were found with chemometric analyses described below. The ISREA baselining procedure with different node sets enabled focusing on specific regions of spectra while omitting variance arising from non-specific Raman shift regions.

### Analysis 1: identifying differences between DKD and IMN

**Figure 2:**
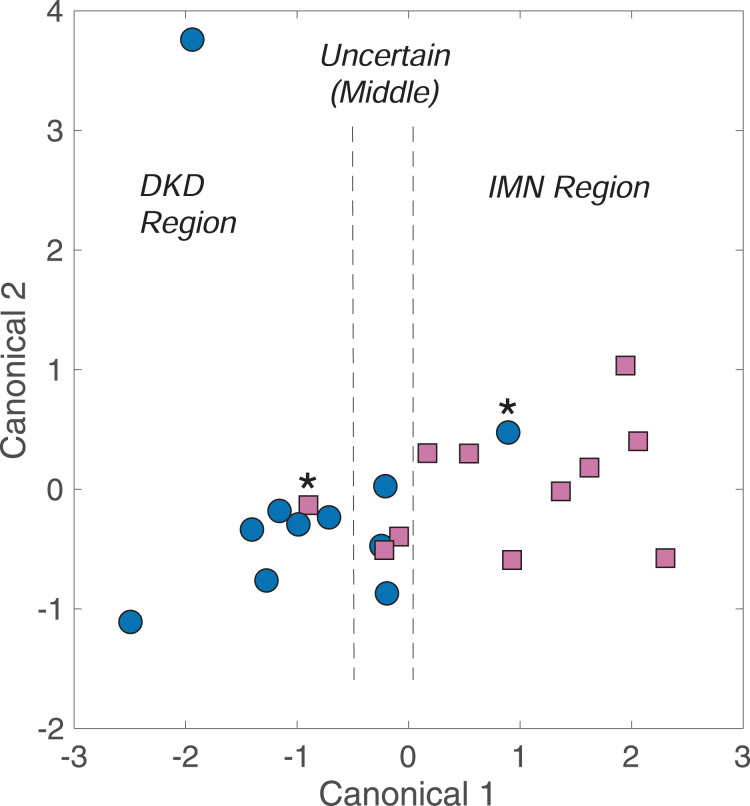
Fingerprinting analysis of DKD and IMN urine samples by DAPC. Samples marked by asterisk (*) signify mis-classifications by the model.

As shown in Fig. 1, spectral differences exist between urine spectra of patient with DKD and IMN. DAPC models were built using these spectra (11 of each), omitting the one patient with both DKD and IMN. To best separate DKD and IMN urine spectra, ISREA nodes at 400, 846, 944, 988, 1,031, 1,196, 1,665, and 1,800 cm^−1^ were used. The results of the leave-one-out analysis reveal prediction metrics of 82% accuracy as well as 82% sensitivity, specificity, PPV, and NPV. Nine (9) of 11 DKD samples and nine of 11 IMN samples were predicted correctly. A cluster analysis from DAPC modeling is shown in Fig. 2. Here, each data point represents a processed Raman spectrum of a urine sample (including scan replicate averaging). Data points clustering near one another indicate spectral similarity over the entire Raman spectrum. Since the entire Raman spectrum is considered in this analysis, this is referred to as spectral (or molecular) fingerprinting. We note that insignificant clustering was observed when using PCA only. However, PCA was used to consolidate the dataset variance (from Raman intensity values at different wavenumbers) into a small number of PCs. The DAPC model was constructed with enough PCs to represent 99% of the dataset variance. In this case of this dataset, this required the top six PCs. DAPC clustering results, shown in Fig. 2, revealed distinct cluster regions for DKD and IMN, with an “Uncertain” region consisting of an overlap of data points. This produced six canonicals in DAPC (*i.e*., axes in Fig. 2). Where ~80% of the samples clustered along Canonical 1, the uncertain region was resolved with other canonicals. Next, the PC and DAPC loadings were analyzed to determine which Raman shifts led to cluster separations. Detailed results (Table S1 and PC and DAPC loadings plots Figs. S1 and S2) are given in the Supplemental Appendix. In summary, major Raman band contributors to both PCA and DAPC cluster separations between DKD and IMN samples included: carbohydrates (specifically glucose) (898, 931, 1,060, 1,105 cm^−1^); collagen, protein, and amide III (890, 1,004, 1,007, 1,237, 1,242, 1,318 cm^−1^); urea (1,002 cm^−1^); phosphatidylinositol (776 cm^−1^); and nucleic acids (826, 1,055, 1,078, 1,318 cm^−1^).

### Analysis 2: distinguishing among all glomerular diseases simultaneously

**Figure 3:**
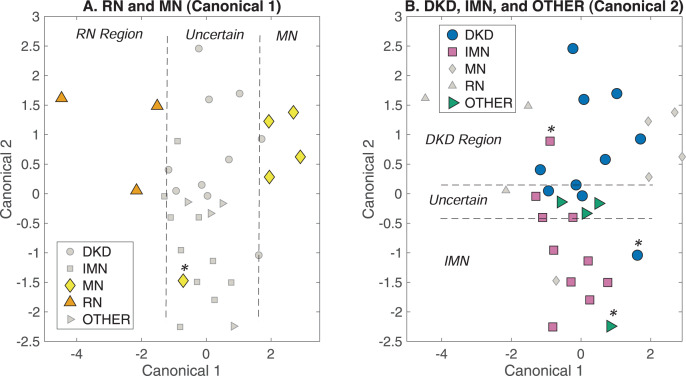
DAPC fingerprinting for resolving glomerular disease Raman urine spectra. (A) Separation of RN and MN along Canonical 1, and (B) resolution of DKD and IMN along Canonical 2. The uncertain region containing DKD, IMN, and other samples were resolved along additional canonicals. Samples marked by asterisk (*) signify mis-classifications by the model.

The patient samples assigned to classes DKD, IMN, MN, RN, and OT (Table 1) were used in chemometric modeling to determine if they could be distinguished from one another through Raman analysis of urine. ISREA nodes of 400, 433, 666, 722, 764, 1,469, 1,479, and 1,800 cm^−1^ were used for spectra baselining. With the five separate classes mentioned above, the random chance accuracy of correct prediction is 20%. The resulting overall prediction accuracy was 46.9%, meaning the DAPC model out-performed random chance assignments. For all individual classes (DKD, IMN, MN, RN, and Other), the sensitivity, specificity, PPV, and NPV values ranged between 40–100% (random chance = 20% here as well). All prediction metrics and actual class predictions for all samples are given in Tables S2 and S3 in the Supplemental Appendix. DAPC fingerprinting plots for this analysis are given in Fig. 3. Here, the separation between DKD and IMN is still apparent but is confounded by the presence of other glomerular diseases. Starting with Canonical 1 (Fig. 3A), both RN and MN were separated from each other and remaining clusters. The DKD, IMN, and Other samples did not separate along Canonical 1 and were included in an “Uncertain” region. This Uncertain region was analyzed along Canonical 2 in Fig. 3B. Here, separation was observed between DKD and IMN, and a new Uncertain region contained 75% of the OT samples, and ~20% of the DKD and IMN samples. These samples were separated along additional canonicals.

### Analysis 3: finding one disease class among all glomerular diseases

Next, a “positive/negative” analysis was conducted among the glomerular disease classes (DKD, IMN, MN, RN, OT). When analyzing a specific class, the dataset was divided into two subclasses, one containing the disease of interest (*i.e*., “positive”) and the others samples comprising other diseases (*i.e*., “negative”). The OT class was not used as a “positive” case because it contains multiple samples with non-specific pathologies, but it was included in the “negative” subclass of glomerular diseases. The DAPC chemometric approach was applied to this scenario, and ISREA nodes were designed for each disease class. Here, the random chance accuracy of correct prediction was 50% (positive/negative). However, this value is also bound by scenarios in which all positive or all negative predictions are returned. In this scenario, the number of positive samples in the dataset is significant. For example, in a dataset with four positive samples and 16 negative samples that returns all negative predictions, the accuracy would be 16/20 = 80%. Likewise, if all positive predictions were returned, this would lead to 4/20 = 20%. Thus, the random chance accuracy is represented here as 50% [20, 80], with the numbers in brackets representing these all-negative and all-positive prediction scenarios. For the hypothetical case described, significant prediction accuracies were considered those >80%. Prediction metrics from leave-one-out cross-validation and the ISREA nodes used for each disease class are given in Table 2. All glomerular disease classes produced a prediction accuracy that exceeded the random-chance accuracy corrected for its prevalence in the dataset.

**Table 2:** ISREA nodes and prediction metrics for detecting one disease among a population of five glomerular diseases (DKD, IMN, MN, RN, OT).

Glomerular disease class	ISREA nodes (cm^−1^)	Random-chance accuracy*	Prediction accuracy	Sensitivity	Specificity	PPV	NPV
DKD	400, 917, 940, 1,058, 1,558, 1,800	50% [35.3, 64.7]	76.5%	75.0%	77.2%	64.3%	85.0%
IMN	400, 510, 1,121, 1,488, 1,618, 1,623, 1,704, 1,800	50% [35.3, 64.7]	82.4%	83.3%	81.8%	71.4%	90.0%
MN	400, 463, 1,460, 1,483, 1,508, 1,681, 1,800	50% [17.7, 82.3]	91.2%	66.7%	96.4%	80.0%	93.1%
RN	400, 1,202, 1,232, 1,271, 1,323, 1,516, 1,717, 1,800	50% [8.8, 91.2]	100%	100%	100%	100%	100%

**Note:**

* The values in brackets [ ] represent the calculated accuracies if either all positive or all negative predictions were returned.

### Analysis 4: detecting DKD among a larger population

The approach in Analysis 3 was repeated for DKD, but the negative (*i.e*., “non-DKD”) subclass consisted of the un-biopsied DM− class, the HEALTHY class samples, IMN samples, the PATH class samples (DM−), and SURINE class samples (Table 1). Since the DM+ and DM− classes are un-biopsied, only the DM− class was included as “non-DKD” because it is highly possible that some of the DM+ class would classify as DKD if biopsied. This produced a total dataset size of 150 samples, with only 11 classifying as DKD. Note, the sample with both DKD and IMN was left-out of the analysis. Thus, the random-chance accuracy for this dataset was 50% [7.3, 92.7]. The actual prediction accuracy was found to be 93.3%, exceeding that for the case of all-negative predictions (92.7%) (Table 3). While the sensitivity of this analysis (36.4%) was less than that obtained when screening against glomerular diseases only (75.0%), the PPV and NPV values of the two analyses differed by ~10%. Based on calculations from this dataset, the NPV suggests that 95.1% of patients who would screen negative for DKD by Raman spectroscopy of urine and Rametrix^®^ analysis would also go on to be found negative for DKD if biopsied.

**Table 3:** Prediction metrics for a dataset containing 11 DKD samples among a population also containing other glomerular diseases, healthy volunteers, Surine™, and un-biopsied DM-patients.

Number of DKD samples included	Total dataset size (Samples)	Random-chance accuracy	Prediction accuracy	Positive* samples predicted correctly (Sensitivity)	Negative** samples predicted correctly (Specificity)	PPV	NPV
11	150	50% [7.3, 92.7]	93.3%	4/11 = 36.4%	136/139 = 97.8%	57.1%	95.1%

**Note:**

* Positive samples here are those with DKD.

** Negative samples are those without DKD.

### Analysis 5: how many un-biopsied patients with DM would screen positive for DKD?

The urine Raman spectroscopy and Rametrix^®^ model from Analysis 4 was used to screen the un-biopsied diabetic patients for the presence of DKD. It was found that 13 of 156 (8.33%) un-biopsied diabetic patients screened positive for DKD with this model.

### Analysis 6: detecting IMN with/without DM among larger populations

An analysis similar to Analysis 4 was performed with the goal of detecting IMN. To do this, the IMN samples were divided into two classes (i) those without DM (IMN/DM−) and (ii) those with DM (IMN/DM+). Next, the IMN/DM- class (six samples) was screened against a larger population containing the DKD, DKD/OT, and DM+ classes (Table 1). No un-biopsied DM- class samples were included since some may be assigned to the IMN/DM− class if biopsied. The total dataset contained 181 samples, with six IMN/DM− samples labeled “positive” and the remainder “negative”. This produced the random chance prediction accuracy of 50% [0.033, 96.7]. Cross-validated prediction results are also shown in Table 4, and a prediction accuracy of 98.4% was achieved, including correct prediction for three of six IMN/DM− samples (50.0% sensitivity). Next, the IMN/DM+ class (six samples) was screened against a larger population containing the following classes: HEALTY, SURINE, PATH, and DM− (Table 1). No un-biopsied DM+ samples were included in the dataset. With IMN/DM+ class samples labeled as “positive” and the remaining “negative”, the random chance prediction accuracy was 50% [0.045, 95.5]. Actual cross-validation results yielded an accuracy of 97.0%, with five of six IMN/DM+ samples (83.3% sensitivity) predicted correctly. Other prediction metrics are given in Table 4.

**Table 4:** Prediction metrics for a dataset containing 6 IMN/DM+/− samples among a population also containing other glomerular diseases, healthy volunteers, Surine™, and un-biopsied DM+/− patients.

Number of IMN samples included and class	Total dataset size (Samples)	Random-chance accuracy	Prediction accuracy	Positive* samples predicted correctly (Sensitivity)	Negative** samples predicted correctly (Specificity)	PPV	NPV
6 IMN/DM−	181	50%	98.4%	3/6 = 50.0%	174/175 = 99.4%	75.0%	98.3%
		[0.033, 96.7]					
6 IMN/DM+	134	50%	97.0%	5/6 = 83.3%	125/128 = 97.7%	62.5%	99.2%
		[0.045, 95.5]					

**Notes:**

* Positive samples here are those with IMN.

** Negative samples are those without IMN.

### Analysis 7: how many un-biopsied patients would screen positive for IMN?

The models from Analysis 6 were used to screen the un-biopsied patients for the presence of IMN. First, the IMN/DM- model was used to screen the un-biopsied non-diabetic patients. Of this population, 1 of 62 (1.6%) screened positive for IMN. Next, the IMN/DM+ model was used to screen the un-biopsied diabetic patients. In this case, 20 of 156 (12.8%) screened positive for IMN.

### Analysis 8: DKD with overlapping pathologies

Of the 12 DKD samples used in this study, three were overlapping with other pathologies: IMN (one sample), segmental glomerulosclerosis (one sample), and RN (one sample). Nine DKD samples had no overlapping pathologies and are classified as “DKD/NO” in Table 1. An analysis was performed to determine if this DKD/NO class could be differentiated from the “PATH” class (Table 1), which contains nine samples in the dataset with varying glomerular pathologies but all DM−. The results are given in Table 5 under “DKD/NO *vs* PATH”, and all metrics were 100%. Next, a repeat of Analysis 1 was performed where the DKD samples with no overlapping pathologies were compared to IMN samples. It was hypothesized that overlapping pathologies may obscure the resolution between DKD and IMN. Results are shown in Table 5 as “DKD/NO *vs* IMN”. In this study, similar metrics (within 5%) were obtained when comparing the DKD *vs* IMN classes in Analysis 1.

**Table 5:** Prediction metrics for the DKD class without overlapping pathologies “DKD/NO” *vs* the PATH and IMN classes.

Disease classes and total dataset size (Samples)	Number of DKD/NO samples included	Random-chance accuracy	Prediction accuracy	Positive* samples predicted correctly (Sensitivity)	Negative** samples predicted correctly (Specificity)	PPV	NPV
DKD/NO *vs* PATH 18	9	50% [50, 50]	100%	9/9 = 100%	9/9 = 100%	100%	100%
DKD/NO *vs* IMN 20	9	50% [42.9, 57.1]	80.0%	7/9 = 77.8%	9/11 = 81.8%	77.8%	81.8%

**Notes:**

* Positive samples here are those with DKD.

** Negative samples are those without DKD.

## Discussion

This proof-of-concept study suggests Raman spectroscopy-based chemometric analysis of urine with Rametrix^®^ can detect the presence of several glomerular pathologies, including DKD, IMN, MN, and RN. In particular, this dataset collected from Veterans at the SVAHCS allowed more thorough investigation of DKD and IMN, and specific spectral signatures (*i.e*., fingerprints) for each. The Raman bands associated with the differences in DKD and IMN fingerprints are largely associated with carbohydrates (particularly glucose), urea, collagen, and other proteins/peptides. While these may be attributed to DM, one half of the IMN samples were from diabetic patients (only one with DKD comorbidity). Among DKD, IMN, MN, and RN renal pathologies, DKD proved the most difficult to resolve among the others (76.5% accuracy), and the admittedly few RN samples of this study were resolved with 100% accuracy (as well as sensitivity, specificity, PPV, and NPV). Additional analyses in this study showed that DKD and IMN urine samples from biopsied patients can, with better than random-chance accuracy, be identified from samples of other un-biopsied CKD patients, diabetic patients who have alternative causes for their kidney disease (as determined by biopsy), healthy volunteers, and the urinalysis control, Surine™. We recognize our dataset as adequately sized for a proof-of-concept study. However, this dataset size limited our validation procedures to leave-one-out analysis (as opposed to k-fold cross-validation) to determine how a urine sample unknown to our models would be classified.

These findings allowed us to build a Rametrix^®^-based urine screening test for DKD and IMN and apply it to the un-biopsied CKD patients enrolled in this study. Of the 156 screened un-biopsied diabetic patients, 13 (8.33%) were found to screen positive for DKD and 20 (12.8%) were found to screen positive for IMN. Of the 62 non-diabetic CKD patients, only 1 (1.6%) screened positive for IMN. Of a population of diabetic CKD patients, it has been reported that about 40% will be positive for DKD if biopsied and another 40% positive for non-DKD pathologies (Souza et al., 2020; Tong et al., 2020). These are higher than our respective 8.33% (for DKD) and 12.8% (for IMN) results reported here. As shown previously, Rametrix^®^ screens can be tailored to favor high sensitivity or specificity by adjusting the number of PCs used to build DAPC models (Huttanus et al., 2020). Our choice of using enough PCs to represent 99% of the dataset variance led to using six PCs or fewer in building the DAPC models of this study. This number is not absolute and can be adjusted for future larger datasets. However, clearly, the DKD model reported here (36.4% sensitivity, 97.8% specificity) favors specificity. This is important as it minimizes false-positive screening tests. In the case of DKD, the goal of a screening test is to identify only those patients in need of a confirmatory biopsy to ensure proper treatment. A false-positive screen may subject a patient to an un-necessary and invasive biopsy that compromises patient comfort and utilizes valuable hospital resources and personnel. The hypothesis is that patients returning a false-negative urine screen will test positive in future screens. Because a Raman spectroscopy-based urine screen is inexpensive and non-invasive, it can be administered to patients regularly.

These preliminary findings, while provocative, need more extensive validation to justify this method of ‘chemometric urinalysis’ as useful in disease discrimination and management. The development of models with greater discriminating ability (particularly improved sensitivity) will result from larger sample sizes and modifications to the model. In particular, the biopsy-confirmed DKD samples used in this study were largely from late-stage CKD patients presenting with significant proteinuria values. More diversity among biopsied DKD samples will lead to improved sensitivity of the screening model and identification of more DKD patients. This is also true of the other renal pathologies. Thus, the Rametrix^®^-based screen could potentially be utilized as a tool to guide decision-making regarding whether to biopsy a patient with DM and in whom there is a suspicion of renal pathology not related to DM.

We recognize limitations of our current study included a relatively small percentage of patients who have had renal biopsies performed (34 biopsied/263 patients = 12.9%). In addition, the patient population of Veterans has an average age of 68 and is largely male (92.4%). It is unclear if/how these results will translate to more diverse patient populations. An in-depth analysis of possible confounding clinical factors (comorbidity, disease severity, management) in the patients within the biopsy group is necessary. Since not all patients with non-DKD pathologies on biopsy have DM, it is possible that the differences detected between DKD and other glomerular pathology group may actually be due to the presence of DM. Some patients with non-DKD pathologies on biopsy did have DM as comorbidity. It will be important to characterize the Raman spectra of non-DKD glomerular pathology in non-diabetic patients in addition to characterizing it in diabetic patients.

Some patients in this study also had evidence of both DKD and other glomerular diseases on biopsy. In these cases, we relied upon the pathologist’s estimation of which was more significant and clinical thinking to determine which was the most significant contributor to the patient’s CKD. It is possible that this limited set of ‘dual’ pathology samples reduced the accuracy of the model and perhaps dual disease can result in unique clustering that shares characteristics of both groups. Additionally, it remains to be seen if the severity of renal dysfunction (*e.g*., decreased eGFR) between the DKD biopsy group and non-DKD biopsy group contributed to any clustering differentiations between the two.

We recognize that we have not determined how the management and severity of DM in individual patients may have affected these results. The effects of day-to-day variability in blood and urine glucose, diet, lifestyle, and efficacy of DM management require further investigation (Senger et al., 2019).

Our existing database of healthy volunteers is predominantly composed of younger healthy adults and is majority female (Senger et al., 2019). This database was from first morning voided samples of patients while the Veteran population of this study (older and majority male), provided random samples throughout the day. Ongoing studies will need to address this disparity, but obtaining urine specimens from healthy volunteers in a Veterans Affairs hospital setting was found challenging.

## Conclusions

In this study, we tested the hypotheses that Raman spectroscopy of urine and Rametrix^®^ chemometric analysis could (i) detect discernable differences in urine samples from patients with DKD and IMN, (ii) distinguish among a variety of glomerular pathologies, and (iii) be used as a urine screening tool among larger populations. Rametrix^®^ was capable of discerning among urine of patients with four different glomerular pathologies in this study, including DKD and IMN. In the future, we intend to increase the sample size of all groups, focusing on patients with biopsy-characterized disease. We will also include correlation of serum creatinine levels, blood glucose, proteinuria, BUN, and eGFR with our Raman spectroscopy results. We speculate that eGFR differences, and the presence of DM as a comorbidity, may be significant factors in differentiating spectra.

In summary, the current study demonstrates the potential utility of Raman spectroscopy and application of chemometric algorithms with Rametrix^®^ for evaluating renal pathologies in CKD patients, based on Raman spectral analysis of urine molecular composition. Ongoing validation of these methods and technologies is underway.

## Supplemental Information

10.7717/peerj.14879/supp-1 Supplemental Information 1 Supplementary Tables.

## Additional Information and Declarations

### Data Availability

The following information was supplied regarding data availability: Senger, Ryan S., Robertson, John L., Kavuru, Varun, & Choudhury, Devasmita. (2022). Raman spectra of urine from patients with diabetes mellitus and other pathologies [Data set]. Zenodo. https://doi.org/10.5281/zenodo.7343559.
